# Anatomical Organization of the Rat Subfornical Organ

**DOI:** 10.3389/fncel.2021.691711

**Published:** 2021-09-06

**Authors:** Amirah-Iman Hicks, Simona Kobrinsky, Suijian Zhou, Jieyi Yang, Masha Prager-Khoutorsky

**Affiliations:** Department of Physiology, McGill University, Montreal, QC, Canada

**Keywords:** glia cells, tanycytes, osmoregulation, angiotensin II, fenestrated blood capillary, blood-brain barrier

## Abstract

The subfornical organ (SFO) is a sensory circumventricular organ located along the anterodorsal wall of the third ventricle. SFO lacks a complete blood-brain barrier (BBB), and thus peripherally-circulating factors can penetrate the SFO parenchyma. These signals are detected by local neurons providing the brain with information from the periphery to mediate central responses to humoral signals and physiological stressors. Circumventricular organs are characterized by the presence of unique populations of non-neuronal cells, such as tanycytes and fenestrated endothelium. However, how these populations are organized within the SFO is not well understood. In this study, we used histological techniques to analyze the anatomical organization of the rat SFO and examined the distribution of neurons, fenestrated and non-fenestrated vasculature, tanycytes, ependymocytes, glia cells, and pericytes within its confines. Our data show that the shell of SFO contains non-fenestrated vasculature, while fenestrated capillaries are restricted to the medial-posterior core region of the SFO and associated with a higher BBB permeability. In contrast to non-fenestrated vessels, fenestrated capillaries are encased in a scaffold created by pericytes and embedded in a network of tanycytic processes. Analysis of c-Fos expression following systemic injections of angiotensin II or hypertonic NaCl reveals distinct neuronal populations responding to these stimuli. Hypertonic NaCl activates ∼13% of SFO neurons located in the shell. Angiotensin II-sensitive neurons represent ∼35% of SFO neurons and their location varies between sexes. Our study provides a comprehensive description of the organization of diverse cellular elements within the SFO, facilitating future investigations in this important brain area.

## Introduction

The central nervous system is separated from the peripheral circulation by the blood-brain barrier (BBB) (Ehrlich, 1885; Lewandowsky, 1909), which is formed by specialized endothelial cells, astrocytes, and pericytes (Tagami et al., 1990). Circumventricular organs are highly vascularized midline structures lacking a complete BBB (Hofer, 1958; Hoffman and Olszewski, 1961) due to the presence of fenestrated vasculature, allowing peripheral circulating factors to penetrate the brain and influence neuronal activity (Bearer and Orci, 1985; Krisch et al., 1987; Shaver et al., 1990; McKinley et al., 2003).

The subfornical organ (SFO), named due to its proximity to the fornix, is one of the sensory circumventricular organs (Dempsey, 1968). Basic anatomical features of the SFO have been documented in several mammalian species, including sheep (McKinley et al., 1983), rabbit (Weindl and Joynt, 1972), cat (Akert et al., 1961; Pfenninger et al., 1969), mouse (Schinko et al., 1971), rat (Spoerri, 1963; Dellmann and Simpson, 1976), and human (Mark and Farmer, 1982). As in other species, rat SFO is located along the anterior wall of the third ventricle, where it occupies the dorsal extremity of the lamina terminalis, extending into the lumen of the third ventricle and adjacent to the choroid plexus (McKinley et al., 2003).

Previous work has shown that the SFO is a heterogeneous nucleus that contains several types of neurons that differ in their pattern of gene expression. Notably, a few subsets of neurons have been associated with specific neural pathways, e.g., excitatory nitric-oxide synthase (nNOS)-positive neurons trigger thirst (Oka et al., 2015; Augustine et al., 2018, 2019; Zimmerman et al., 2019). In addition, subpopulations of SFO neurons express receptors for hormones associated with regulating energy metabolism, such as amylin (Sexton et al., 1994; Pulman et al., 2006; Smith et al., 2009), ghrelin (Pulman et al., 2006), leptin (Smith et al., 2009), cholecystokinin (Ahmed et al., 2014), neuropeptide Y (Kishi et al., 2005), and glucagon-like peptide 1 (Göke et al., 1995), as well as molecules involved in neuroimmune interactions, such as lipopolysaccharide receptor (Lacroix et al., 1998), prostaglandins (Matsumura et al., 1990; Zhang and Rivest, 1999), interleukin 1b and interleukin 6 (Takahashi et al., 1997), and TNFα (Simpson and Ferguson, 2017). Moreover, SFO neurons express nitric oxide synthase (Jurzak et al., 1994; Alm et al., 1997), relaxin receptors (Burazin et al., 2005), vasopressin V1 receptor (Phillips et al., 1988), atrial natriuretic peptide (Quirion et al., 1984; Mendelsohn et al., 1987), estrogen receptor (Somponpun et al., 2004), prolactin receptor (Kamesh et al., 2018), galanin receptor (O’Donnell et al., 1999), and somatostatin (Patel et al., 1986), as well as ion channels such as TRPV1 (Mannari et al., 2013), Na_x_ (Hiyama et al., 2004; Nehmeì et al., 2012), and classical neurotransmitter receptors such as glutamate, GABA, and acetylcholine (McKinley et al., 2003). Although it remains unknown whether the expression of these molecules defines distinct neuronal subpopulations within the SFO, these findings are consistent with the involvement of this nucleus in a myriad of centrally regulated homeostatic processes, including energy metabolism and food anticipatory activity (Pulman et al., 2006; Smith and Ferguson, 2010), thirst (Hollis et al., 2008), reproduction (Summerlee et al., 1987; Sunn et al., 2002), immune responses (Takahashi et al., 1997), cardiovascular regulation (Cancelliere and Ferguson, 2017; Jeong et al., 2019; Rossi et al., 2019), and systemic osmoregulation (Oldfield et al., 1994).

One of the best-characterized functions of the SFO is its role in mediating dipsogenic and pressor effects of circulating angiotensin II (Ang II) (Bickerton and Buckley, 1961; Simpson and Routtenberg, 1973; Hoffman and Phillips, 1976; Simpson et al., 1978; Ferguson and Bains, 1997). Neurons in this area express Ang II type 1 (AT1) receptors (Song et al., 1992; Allen et al., 2001). Ang II causes an increase in action potential firing rate of SFO neurons (Felix and Akert, 1974; Felix, 1976; Felix and Schlegel, 1978; Mendelsohn et al., 1984; Allen et al., 1988; McKinley et al., 1992), thereby stimulating neural pathways involved in drinking, pressor response, and salt appetite *via* its projections to the bed nucleus of the stria terminalis, the median preoptic, and supraoptic and paraventricular nuclei (Simpson et al., 1978; Weisinger et al., 1990; Oldfield et al., 1994; Johnson et al., 1999; Fitts et al., 2005; Hollis et al., 2008).

Subfornical organ is also an important osmoregulatory region, containing neurons sensitive to hypertonic NaCl or mannitol (Oldfield et al., 1994; McDonald et al., 1998; Caston-Balderrama et al., 1999; Anderson et al., 2000). Ang II and osmosensitive neurons of the SFO contribute to the regulation of body fluid homeostasis *via* axonal projections to many central homeostatic structures, including the hypothalamic paraventricular and supraoptic nuclei, the median preoptic nucleus, and thalamic neurons projecting to frontal thirst areas (Oldfield et al., 1994; Hollis et al., 2008). These projections regulate the activity of magnocellular neurosecretory neurons releasing antidiuretic hormone vasopressin (Oldfield et al., 1994), as well as contribute to the regulation of the activity of pre-autonomic neurons associated with cardiovascular control (Cancelliere et al., 2015; Cancelliere and Ferguson, 2017). In addition, SFO has extensive afferent and efferent neural connections to multiple brain areas within the hypothalamus as well as the brain stem, thalamus, cortex, and other regions (McKinley et al., 2003, 2019).

As in other circumventricular organs, SFO harbors several unique non-neuronal cell populations including specialized endothelial cells containing fenestrations (Sposito and Gross, 1987; Shaver et al., 1990) with extended perivascular space surrounded by basement membranes (Morita et al., 2016) and distinct glia cells called tanycytes (Horstmann, 1954; Krisch et al., 1978; Langlet et al., 2013b).

Most previous functional studies on the SFO have focused on investigating the responsiveness of neurons to humoral factors and the underlying neuronal circuitry, as well as the involvement of different neuronal subpopulations in the regulation of a broad range of physiological functions related to water and salt balance, thirst, and salt appetite (McKinley et al., 2019). However, how these neuronal populations are organized in relation to other cellular elements within the SFO (e.g., glia, vasculature) is ill defined. In this work, we studied the anatomical organization of the SFO in male and female rats by analyzing BBB permeability, as well as the organization of fenestrated and non-fenestrated vasculature, neurons, glia cells, ependymocytes, tanycytes, and pericytes. We also characterized the distribution of neurons activated in response to systemic administration of AngII and hypertonic NaCl solution.

## Materials and Methods

### Animals

Adult male and female Wistar rats (350–400 g, Charles River Laboratories, Saint-Constant, QC, Canada) were used in this study. Animals were housed on a 12 h:12 h light:dark cycle and all animals were treated in strict accordance with the guidelines outlined by the Canadian Council on Animal Care. All experiments adhered to the Animal Use Protocol approved by the Comparative Medicine and Animal Resources Center of McGill University. Vaginal smearing was used to track the estrous cycle of female rats, and female rats were used on the estrus day of their breeding cycle. Brains from both sexes were analyzed. No differences in the morphology and distribution of various cell types within the SFO were detected between males and females, and the examples show brain sections from males, unless specified.

### Immunohistochemistry

Rats were anesthetized with isoflurane and perfused transcardially with 10 ml phosphate-buffered saline (PBS) followed by 250 ml of 4% paraformaldehyde (PFA) in PBS at room temperature. Brains were extracted and post-fixed by immersion for 48 h in 4% PFA in PBS. A vibratome was used to cut 50 μm-thick serial sections in coronal and sagittal planes.

Sections were blocked for 1 h at room temperature with 10% normal goat serum in 0.3% Triton-X in PBS. After blocking, sections were incubated for 24–48 h at 4°C with primary antibodies. Following three washes, sections were incubated for 1.5 h with fluorescently labeled secondary antibodies and DAPI (1:5,000, Invitrogen, CA, United States). Sections were then washed and mounted on glass slides using Prolong Gold Antifade mounting media (Invitrogen). All images were collected using confocal microscopy. Stereotaxic coordinates mentioned in the text were derived from the 7th edition of the rat brain atlas published by Paxinos and Watson (2018).

### Antibodies

The following primary antibodies were used: Mouse monoclonal anti-RECA1 (Abcam, ab9774, 1:100) that recognizes a cell surface antigen expressed on the surface of rat endothelial cells. RECA1 is a pan-endothelial marker and labels both fenestrated and non-fenestrated vasculature. Fenestrated blood vessels were labeled with rabbit polyclonal antibody against plasmalemmal vesicle-associated protein 1 (PV1) provided by Dr. Philippe Ciofi (INSERM, Bordeaux, France, 1:250) (Ciofi et al., 2009). Tanycytes and ependymocytes were labeled with chicken polyclonal antibody against vimentin (Millipore, AB5733, 1:5,000). Vimentin is an intermediate filament that in adult tissue is primarily expressed by tanycytes and ependyma cells (Franke et al., 1978). Astrocytes were labeled with rabbit polyclonal GFAP (glial fibrillary acidic protein) antibody (Abcam, ab7260, 1:1,000), neurons with NeuN (Hexaribonucleotide Binding Protein-3a) guinea pig polyclonal antibody (Millipore, ABN90, 1:500), NG2 glia and pericytes with rabbit polyclonal antibody against the chondroitin sulfate proteoglycan NG2 (Millipore, AB5320, 1:500) and goat polyclonal anti-CD13 (R&D systems, AF2335, 1:100), and c-Fos with rabbit polyclonal antibody (SYSY, 226 003, 1:500). Secondary antibodies were Alexa Fluor-Conjugated (488, 568, and 647 nm; Invitrogen, 1:500).

### Evans Blue Injections

Subfornical organ boundaries were assessed using Evans Blue (EB) as previously described (Gregersen and Rawson, 1943; Wolman et al., 1981; Prager-Khoutorsky and Bourque, 2015). Rats anesthetized with isoflurane were injected intravenously *via* the femoral vein with 1 ml of 1% EB dissolved in saline (Sigma, E2129, 0.25 ml/100 g). After 30 min, rats were transcardially perfused with 20 ml of PBS to wash out the dye from the circulation, and then the brains were extracted and fixed for at least 48 h by immersion in 4% PFA in PBS. Serial 50 μm sections were cut and mounted on slides, and EB fluorescence was imaged at 647 nm using confocal microscopy.

### Angiotensin II Stimulation

For the systemic stimulation with Angiotensin II (Ang II), rats were briefly anesthetized with isoflurane. AngII (A9525, MilliporeSigma, MA, United States) was dissolved in 10 ml saline for a stock concentration of 0.5 mg/ml. Rats were subcutaneously injected with 2 mg/kg of Ang II (Saxena et al., 2015), and returned to their home cage for 1.5 h without access to water. Animals were then perfused with PFA and brains were processed for immunohistochemistry to visualize c-Fos immunoreactivity.

### Hypertonic Stimulation

Rats were stimulated with hypertonic NaCl solution as described previously (Prager-Khoutorsky and Bourque, 2015). Rats were briefly anesthetized with isoflurane and received a subcutaneous injection of 20 ml/kg of isotonic (0.15 M NaCl) or hypertonic (1 M NaCl) saline. Lidocaine (0.25 ml) was added to the injection solutions, and the injection volume was divided between the left and right flanks. After the injection, rats were returned to their home cage without access to water. 1.5 h later, rats were transcardially perfused with PFA, processed for immunohistochemistry to visualize c-Fos. Blood was collected from the right atrium prior to the insertion of the perfusion line into the left ventricle. Blood samples were placed at 4°C for 1 h and then centrifuged for 5 min at 1,200 *g* at 4°C. Serum samples were collected and assayed for plasma osmolality in triplicates.

### Image Acquisition and Analysis

All images were collected using LSM 880 Apo 20x/0.8 or 63x/1.40 oil objectives (Zeiss AG, Oberkochen, Germany). Confocal z-stacks were generated by acquiring 20–25 optical sections with 1.5–2 mm step at 20x and 80–90 optical sections with 0.2–0.4 mm step at 63x. For the quantification of EB, the intensity of EB fluorescence was subtracted from the background intensity measured in the ventral hippocampal commissure (vhc), giving a relative intensity value. For the quantification of fraction of cells expressing c-Fos protein, coronal sections from rostral, medial, and caudal SFO were analyzed as z-stacks containing 10 optical sections 2 mm apart. A fraction of c-Fos and NeuN-double positive cells was calculated out of total NeuN-positive cells for each section. Three coronal sections representing the rostral, medial and caudal regions of SFO were analyzed to obtain the average number of c-Fos-positive cells per brain. All images were processed and analyzed using Fiji (NIH), Imaris (Oxford Instruments), and figures assembled using Adobe Illustrator (Adobe Inc.).

### Statistics

All analyses were performed using Prism 9 (GraphPad Software). Results are reported as mean plus or minus standard error of the mean (±SEM). Data were assessed for normality (Shapiro–Wilk test) and if the data did not satisfy these conditions, a non-parametric method was used. Data of two groups were compared by a two-tailed, unpaired, parametric Student’s *t* test. For parametric multiple comparisons, we used one-way ANOVA followed by between group comparisons using Tukey’s *post hoc* test, or a two-way ANOVA followed by between-group multiple comparison Tukey’s or Sidak’s *post hoc* test. Kruskal–Wallis with *post hoc* Dunn’s test was used for non-parametric multiple comparisons analysis. The significance level was set at *p* < 0.05; ^∗^*p* < 0.05, ^∗∗^*p* < 0.001, ^****^p < 0.0001.

## Results

### BBB Permeability Within the SFO

**FIGURE 1:**
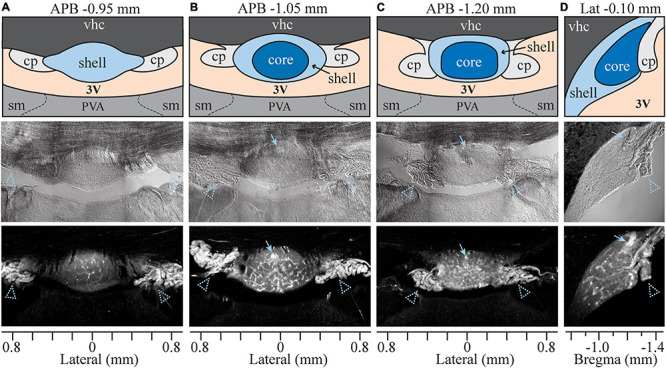
Evans Blue analysis in sagittal and coronal planes. Four sets of column panels show the organization of the SFO and surrounding anatomical structures in coronal plane through the rostral **(A)**, medial **(B)** and caudal **(C)** SFO, and in mid-sagittal plane **(D)**. Anterior-posterior bregma (APB) and lateral coordinates (for the sagittal plane) are indicated above each column. Axes at the bottom of each column show lateral **(A–C)** or bregma **(D)** coordinates in millimeters. Middle and bottom rows show brightfield and Evan Blue (EB) fluorescence respectively, and the top row displays schematic diagrams depicting the SFO and surrounding anatomical structures corresponding to micrographs below. Blue arrows indicate subfornical artery and arrowheads choroid plexus. 3V, third ventricle; vhc, Ventral hippocampal commissure; cp, choroid plexus; PVA, anterior nucleus of paraventricular thalamus; sm, stria medullaris.

The SFO is a small, ovoid structure that bulges into the midline anterior wall of the third ventricle (Figure 1). SFO is divided into two major subdivisions based on morphological, functional, and neuroanatomical criteria: a rostro-dorsal “outer shell” and a central “ventromedial core” (McKinley et al., 2003; Figure 1). Some studies also divide the outer shell into the dorsal and ventral shell areas (Sposito and Gross, 1987; Shaver et al., 1990). To assess the permeability of the BBB within the different subdivisions of the SFO, we used Evans Blue (EB), a vital dye which has classically been used to study the permeability of the BBB (Wolman et al., 1981). Eight rats were injected intravenously with 1% EB as described previously (Prager-Khoutorsky and Bourque, 2015). EB binds to albumin and selectively permeates the brain through fenestrated capillaries in areas lacking a complete BBB. The distribution of EB fluorescence was analyzed in serial coronal and sagittal sections of the SFO (Figure 1). EB fluorescence was detected within the ventral bulge of the lamina terminalis between anterior-posterior bregma (APB) coordinates −0.8 and −1.4 mm, delineating the boundaries of the SFO. The dye did not penetrate the adjacent ventral hippocampal commissure (vhc) located dorsally, thus, creating a clear demarcation between the SFO and the surrounding tissue. Notably, the intensity of the EB fluorescent signal was not uniform throughout the SFO. EB fluorescence in the rostral part of the SFO (around APB coordinates −0.8 to −0.95 mm) is mostly localized to the dorsal portion of the SFO (Figure 1A). Caudal regions of SFO (APB, −1.05 to −1.20 mm) show stronger EB fluorescence than the rostral part (*p* < 0.021, Supplementary Figure 1A) that is distributed more evenly throughout the dorsoventral plane (Figures 1B,C). Notably, there is a stronger EB signal in the caudal portion of the SFO, corresponding to the location of the subfornical artery (Figures 1B–D, arrowhead). These variations in the EB fluorescence likely reflect the regional differences in the BBB permeability within the SFO, and may be due to local differences in the type of vasculature present in each region (Sposito and Gross, 1987).

### Distribution of Fenestrated and Non-fenestrated Vasculature

The major blood supply to the SFO originates from the subfornical artery, which branches from the anterior cerebral artery. Within the SFO, the subfornical artery further branches to form a dense capillary plexus (Spoerri, 1963) that contains fenestrated and non-fenestrated vessels (Sposito and Gross, 1987). The capillaries drain rostrally and dorsally into large medial septal veins, which project dorsally and drain into the system of the great vein of Galen (Spoerri, 1963; McKinley et al., 2003). To analyze the distribution of fenestrated and non-fenestrated vasculature within the SFO, we performed double immunostaining for the pan-endothelial marker RECA-1 and fenestrated vasculature marker plasmalemmal vesicle-associated protein 1 (PV1). PV1 is a membrane-bound glycoprotein that is found in the pores of fenestrated vessels, forming homodimers that constitute the fibril network spanning fenestral diaphragms, and thus are present exclusively in the fenestrated endothelium (Bearer and Orci, 1985; Ciofi et al., 2009).

**FIGURE 2:**
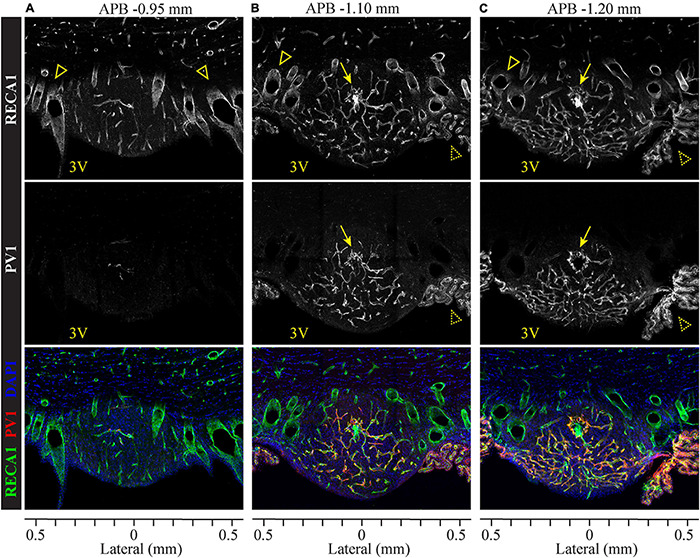
Organization of fenestrated and non-fenestrated vasculature in the SFO analyzed in coronal plane. Distribution of immunolabeled markers for fenestrated and non-fenestrated endothelium in rostral **(A)**, medial **(B)** and caudal **(C)** SFO sections. Pan-endothelial marker RECA1 (rat endothelial cell antigen) is immunostained to visualize all vasculature (white in top panels, green in bottom panels), plasmalemmal vesicle-associated protein 1 (PV1) labeling shows fenestrated vessels (white in middle panels, red in bottom panels), and DAPI (blue, bottom sections). Anterior-posterior bregma (APB) are indicated above and lateral coordinates are shown under each column. PV1-positive fenestrated capillaries are abundant in the medial and caudal sections **(B,C)**, but almost not found in the rostral SFO section **(A)**. Also note the absence of PV1 staining in the subfornical artery (arrows) and septal veins (open arrowheads). Choroid plexus (dotted arrowheads) contains PV1-positive fenestrated vasculature.

As illustrated in Figure 2, SFO comprises a high density of vasculature found throughout the SFO. Lateral and dorsal portions of the outer shell contain small capillaries but are mostly occupied by large blood vessels which span the SFO in the rostro-caudal axis (Figures 2A–C and Supplementary Video 1). Notably, the rostral part of the SFO lacks fenestrated capillaries (Figure 2A), while the rostro-caudal ventromedial core region features an extensive network of thin fenestrated capillaries forming multiple sinusoids and capillary loops (Spoerri, 1963; Duvernoy and Risold, 2007), some of which extend into the ventral shell toward to the ventricular surface of the SFO (Figures 2B,C and Supplementary Video 1). This basic vascular organization is consistent across the rostro-caudal dimension of the SFO. Importantly, the large blood vessels located in the dorsolateral outer shell lack PV1 labeling and therefore are non-fenestrated, while small capillaries located in the ventromedial core are enriched with the marker of fenestration, PV1 (Figure 2, middle panel). These observations are consistent with previous studies analyzing the distribution of different classes of endothelial cells within the SFO using electron microscopy (Dempsey, 1968; Sposito and Gross, 1987; Shaver et al., 1990; Ciofi et al., 2009). The presence of the fenestrated endothelium primarily in the medial to caudal aspects of the SFO is consistent with our observation that this part of the SFO shows stronger EB fluorescence and thus features a leakier BBB.

Interestingly, small capillaries located in the core of the rostral portion of the SFO are lacking PV1 (Figure 2A and Supplementary Video 1). In addition, we found that the subfornical artery does not express PV1, suggesting that the main artery entering into and supplying the blood to the SFO is non-fenestrated (Figures 2B,C, arrows), while the capillaries that branch from the major artery create a network of fenestrated vessels (Supplementary Video 1).

### Glial Cells, Ependymocytes, Tanycytes, and Pericytes

**FIGURE 3:**
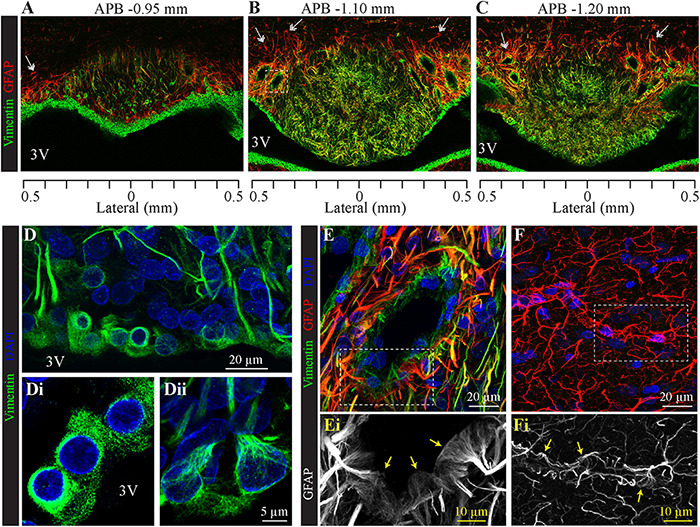
Distribution of SFO ependymocytes, tanycytes, and astrocytes in the coronal plane. Immunohistochemical staining of glial fibrillary acidic protein (GFAP, red) and vimentin (green) in serial coronal sections of SFO corresponding to APB coordinates –0.95, –1.10, and –1.20 mm, respectively **(A–C)**. Note GFAP-positive classical stellate astrocytes are only present in the lateral shell of the SFO (double arrow). **(D)** High magnification images of the section in panel **(B)** showing the ventricular wall created by cell bodies of ependymocytes **(Di)** and tanycytes **(Dii)**, (vimentin green, DAPI blue). **(E,F)** Images of non-fenestrated blood vessels located in the SFO [**(E)**, magnification of area outlined by dotted square in panel **(B)**], and outside the SFO [**(F)**, cortex], labeled for GFAP (red), vimentin (green) and DAPI (blue). Note that non-fenestrated vessels in the SFO are contacted by GFAP-positive and vimentin-positive processes, while cortical vessels are lacking vimentin processes. Areas outlined by dotted lines in panels **(E,F)** are magnified below illustrating GFAP-positive end feet contacting SFO **(Ei)** and cortical **(Fi)** non-fenestrated blood vessels.

**FIGURE 4:**
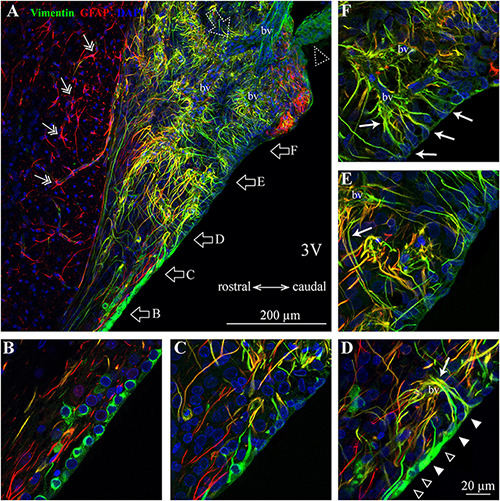
Distribution of SFO ependymocytes, tanycytes and astrocytes in the sagittal plane. **(A)** A low magnification confocal image shows the distribution of vimentin (green), glial fibrillary acidic protein (GFAP, red), and DAPI in a mid-sagittal SFO section (lateral coordinate –0.10 mm). Note GFAP-positive classical stellate astrocytes are only present outside the SFO (double arrow). **(B–F)** Higher magnification images showing ventral portions of the SFO corresponding to areas in panel **(A)** (open arrows). The ventricular wall of the SFO created by ependymocytes in the rostral part **(B)**, which are gradually replaced by tanycytes [**(D)**, ependymocytes open arrowheads, tanycytes full arrowheads]. The caudal part of the SFO ventricular wall is created by cell bodies of tanycytes **(E,F)**. Note tanycytic processes projecting toward capillaries [white arrows in panels **(D–F)**]. Note subfornical artery entering from the caudal part of the SFO and passing through the center of the nucleus (open dotted arrow). The most caudal aspect of the SFO is connected to the choroid plexus (dotted open arrowhead). 3V, third ventricle; bv, blood vessel.

To analyze the distribution of glial cells, we immunolabeled the intermediate filament protein GFAP (glial fibrillary acidic protein), the classical marker of astrocytes (Bignami et al., 1972). As illustrated in Figures 3, 4, GFAP is highly expressed in the processes of glial cells throughout the SFO. Interestingly, the intensity of the GFAP signal is significantly higher in the glia processes located in the dorsolateral shell of the SFO. The most prominent GFAP signal overlaps with the non-fenestrated large blood vessels, which are surrounded by a dense network of thick GFAP-positive processes (Figures 3A–C,E). These processes are present throughout the rostro-caudal axis of the SFO. While GFAP is commonly used as a marker of astrocytes, previous studies have shown that subpopulations of tanycytes also express this protein, as well as an additional intermediate filament molecule vimentin (Leonhardt et al., 1987; Redecker et al., 1987; Robins et al., 2013). Moreover, vimentin, but not GFAP, is also expressed by ependymocytes, which are cuboidal cells lining ventricular walls outside the CVOs (Knowles, 1972; Mathew, 2008). Astrocytes can be identified as cells expressing GFAP and lacking vimentin as illustrated by an example of GFAP-positive and vimentin-negative astrocyte featuring a classical stellate morphology and located in the lateral shell (Figures 3A–C, double arrows), and outside the SFO in the ventral hippocampal commissure (Figure 4A, double arrows). Therefore, in addition to analyzing the expression of cellular markers, the identification of glial cell types requires a consideration of their morphology and position. Notably, ependymocytes and tanycytes are normally restricted to the lining of the cerebral ventricles (Langlet et al., 2013b; Figures 3A–C, 4A). Vimentin-positive cells lining the third ventricle in the most rostral part of the SFO display a classical ependymocyte morphology (Figures 3D, 4A,B), namely, a cuboidal shape organized as a single layer of cells displaying intense vimentin staining surrounding a prominent round, centrally located nucleus and lacking GFAP (Figure 4B).

We found regional differences in the type of cells lining the ventricular wall of SFO. While the ventricular wall is primarily occupied by ependymocytes in the rostral portion of SFO, cells lining the ventricular wall in the medial and caudal aspects of the SFO express much weaker vimentin signal (Figures 3A, 4A–D). The vimentin-positive cells lining the ventricle in the ventral part of the SFO exhibited the typical features of tanycytes, namely, an elongated, oval soma and vimentin-positive apical processes extending into the parenchyma (Figures 3Dii, 4D–F). This transition from ependymocytes to tanycytes is illustrated by analyzing sagittal sections of SFO, showing a continuous population of ependymocytes on the rostral pole of the SFO (Figure 4B) that are replaced by tanycyte cell bodies in the more caudal region (Figures 4D–F).

Tanycytes feature vimentin-positive apical processes that extend into the parenchyma and create an interweaved network that occupies the ventromedial core of the SFO (Figures 3A–C, 4A), and projects toward capillaries within the SFO (Figures 4D–F). Interestingly, while fenestrated capillaries located within the ventromedial core of the SFO are mostly contacted by tanycyte processes which express high levels of vimentin and low levels of GFAP (Figures 4B–F), large non-fenestrated vessels located in the dorsolateral core of the SFO are mostly contacted by the GFAP-positive and vimentin-negative astrocytic processes (Figures 3B,C,E), creating a classical endfeet structure on the non-fenestrated vessels (Figure 3Ei). Notably, detailed examination of these large non-fenestrated vessels reveals that they also encased by vimentin-positive processes (putative tanycytes) (Figure 3E). This is in contrast to non-fenestrated capillaries located outside CVOs which are lacking vimentin-positive processes (Figure 3F).

**FIGURE 5:**
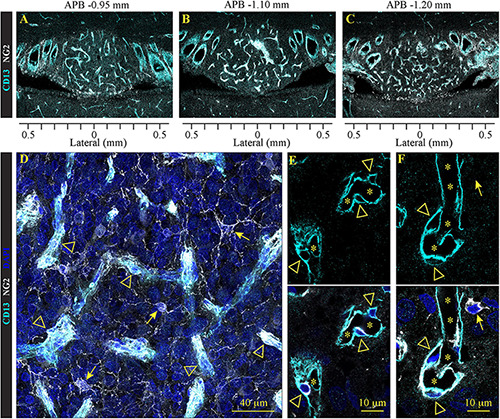
Distribution of pericytes and NG2 glia in the SFO analyzed in the coronal plane. Immunohistochemical staining of pericytes and NG2 glial labeled with NG2 (white), pericyte-specific marker CD13 (cyan), and DAPI (blue) in serial coronal sections of SFO corresponding to APB coordinates –0.95, –1.10 and –1.20 mm **(A–C)**. **(D–F)** High magnification images of pericytes (arrowheads) and NG2 glia (arrows) in close proximity to the SFO vasculature. Stars in panels **(E,F)** indicate the lumen of the vessels.

In addition to astrocytes, ependymocytes, and tanycytes, SFO is comprised of two distinct types of non-neuronal cells: pericytes and NG2-glia cells (Figure 5). Pericytes are mural cells that extend their processes to wrap around and communicate with vascular endothelial cells (Stallcup, 2018). They are an essential component of the neurovascular unit and are involved in the regulation of the BBB (Zheng et al., 2020). NG2 glia cells, also called oligodendrocyte precursor (OPCs) or polydendrocytes (Nishiyama et al., 2002), have the capacity to divide and give rise to mature oligodendrocytes throughout lifespan. Recent studies suggest that these NG2 glia can interact with neurons and other glia cells, and differentiate into neurons and astrocytes (Du et al., 2020). While both pericytes and NG2 glia express the protein NG2 (Chondroitin sulfate proteoglycan 4) (Stallcup, 2018; Du et al., 2020), only pericytes express aminopeptidase N/CD13. Moreover, these cell types feature very distinct morphology: NG2 glia have a classical branched astrocyte shape, while pericytes are ovoid cells located in close proximity to the endothelium, creating a scaffold around capillaries. We found that NG2 glia are scattered ubiquitously across the SFO, as well as outside the nucleus (Figure 5). NG2 glia morphologically resemble stellate protoplasmic astrocytes located outside the SFO (Figures 3F, 4A).

In addition, we detected a network of NG2/CD13-positive pericytes creating a dense scaffold wrapping around the capillary network located in the ventromedial core (Figures 5A–C). These cells feature a typical pericyte morphology with a small oblong cell body and processes extending along the capillaries (Figures 5D–F).

### Distribution of Neurons in the SFO

**FIGURE 6:**
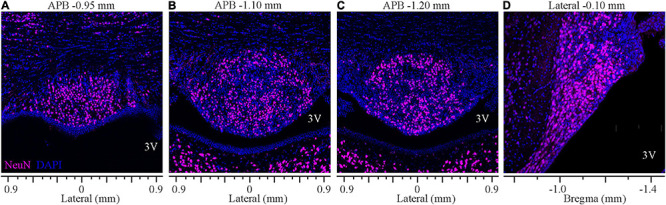
Distribution of neurons in the SFO analyzed in coronal and sagittal planes. Immunohistochemical staining of neurons (NeuN, magenta) and DAPI (blue) of SFO in serial coronal sections of SFO corresponding to APB coordinates –0.95, –1.10, and -1.20 mm **(A–C)** and the mid-sagittal plane corresponding to the lateral coordinate –0.10 mm **(D)**.

Analysis of staining for the neuronal marker NeuN revealed that the ventromedial part of the SFO comprises densely packed neurons bordered dorsally by the ventral hippocampal commissure that lacks neuronal cell bodies, thus, creating a clear demarcation between the SFO and the surrounding tissue (Figure 6). NeuN-positive neuronal cell bodies were homogeneously distributed throughout the nucleus, and dense populations of neurons were observed in both the outer shell and the ventromedial core. However, the overall neuronal density was significantly higher at the medial and caudal aspect of the SFO (Figures 6C,D; *p* < 0.035, Supplementary Figure 1B). Notably, the caudal region of the most ventral part of the SFO, extending into the lumen of the third ventricle and adjacent to the choroid plexus, displayed a lower density of neurons. This area is occupied by dense GFAP-positive processes (Figure 4A), marking the site where the choroid plexus is attached to the caudal surface of the SFO and where large blood vessels surrounded by the arachnoid tissue connect these two regions (Spoerri, 1963).

### Distribution of Neurons Responsive to Systemic Ang II

**FIGURE 7:**
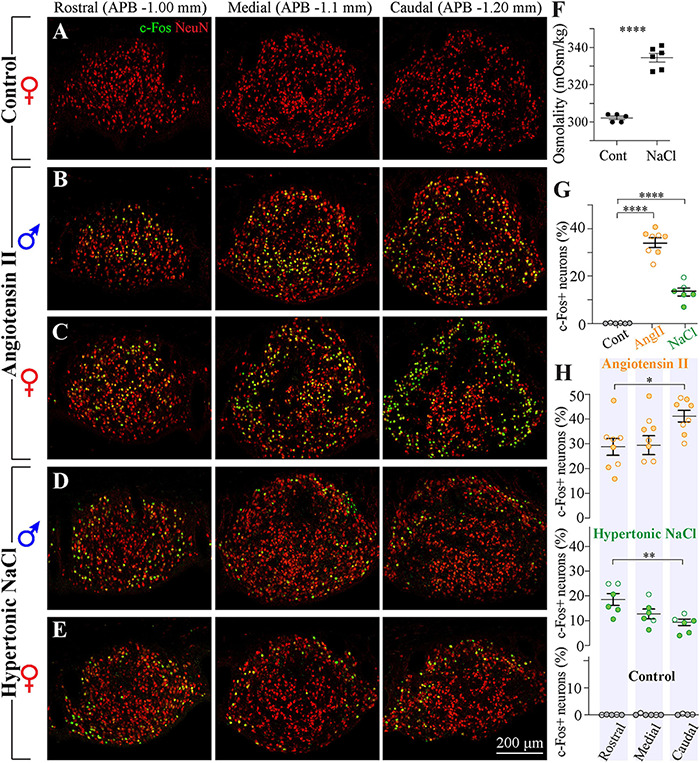
Distribution of neurons responsive to Ang II and hypertonic NaCl stimulation in SFO from male and female rats. **(A–E)** Serial coronal sections representative of the rostral, medial, and caudal SFO regions (APB –1.0, –1.1, and –1.2 mm, respectively) immunolabeled for NeuN (red) and c-Fos (green). Brain sections were derived from rats sacrificed 1.5 h after injection of control isotonic saline [**(A)**, female], 2 mg/ml Ang II [**(B)** male, **(C)** female], or 1 M NaCl [**(D)** male, **(E)** female]. **(F)** Plots show mean ± SEM blood osmolality 1.5 h following the injection of 1 M NaCl or isotonic saline. **(G)** Plots show mean ± SEM percentage of c-Fos positive neurons in different conditions. Each circle represents an average percentage determined by analysis of a rostral, a medial and a caudal SFO section from each rat [*n* = 6 control (isotonic saline), *n* = 8 Ang II, *n* = 6 1 M NaCl]. **(H)** Distribution of c-Fos-positive neurons in the rostro-caudal axis of the SFO in response to injection of 2 mg/ml Ang II (top panel), 1 M NaCl (middle panel), and control isotonic saline (bottom panel). Plots show mean ± SEM percentage of c-Fos-positive neurons analyzed in rostral, medial and caudal regions (APB –1.0, –1.1, and –1.2 mm, respectively). Full circles represent the data from male and open circles from female rats. *p < 0.05, **p < 0.01, and ****p < 0.0001.

Previous *in vitro* electrophysiological studies have reported that a large fraction of SFO neurons is activated by peripherally circulating Ang II (Cancelliere and Ferguson, 2017; Paes-Leme et al., 2018). *In vivo* studies using the expression of the immediate early gene *c-fos* have shown that neurons responsive to intravenous administration of Ang II are distributed throughout the SFO (McKinley et al., 1992), yet previous studies evaluating the expression of the AT1 mRNA using *in situ* hybridization, as well as binding of intravenously injected fluorescently-labeled Ang II, suggest that the density of AT1 receptors is higher in the ventromedial core of the SFO and lower in its outer shell (Lenkei et al., 1995; McKinley et al., 1998; Giles et al., 1999; Allen et al., 2001). These findings are commonly illustrated by a single coronal brain section typically taken through the more rostro-caudal portion of the SFO of male animals. To examine the distribution of Ang II-sensitive neurons throughout the SFO, male and female rats received a subcutaneous injection of either 2 mg/kg of Ang II (four males and four females) or control saline (four males and two females), and the distribution of c-Fos immunoreactivity was examined in serial coronal sections taken throughout the entire SFO. As illustrated in Figure 7, c-Fos-positive neurons were found along the entire rostro-caudal extent of the nucleus. The expression of c-Fos was specifically triggered by Ang II administration, rather than other stimuli, because control animals injected with saline did not show any c-Fos-positive staining (Figure 7A). Consistent with previous reports, in the rostral part of the SFO, these cells were mainly located in the ventromedial core (Figures 7B,C). This pattern of localization was similar in male and female rats. Moreover, the total number of c-Fos positive neurons was not different between male and female rats treated with Ang II (*p* = 0.37, Supplementary Figure 1C). However, in the medial and caudal portions of the SFO, the c-Fos-positive neurons were found in both the ventromedial core and the outer shell (Figures 7B,C). Importantly, our data from female SFO suggest that in the caudal region, Ang II-responsive neurons are located in both the outer shell and the ventromedial core (Figure 7C). Moreover, we found that in females stimulated with systemic Ang II, the majority of outer shell neurons express c-Fos, while only a small fraction of neurons express c-Fos in the ventromedial core. In addition, the Ang II-responsive neurons in the ventromedial core show a much weaker c-Fos signal then neurons located in the outer shell (Figure 7C). These findings suggest that while in the rostral and medial portions of the SFO the distribution of the Ang II-responsive neurons is similar in both sexes, the caudal part of the SFO contains significantly more Ang II-sensitive neurons in the outer shell in female than in male rats (*p* < 0.023, Supplementary Figure 1D), implying that this region contains sexually dimorphic neuronal populations.

Analysis of co-localization between c-Fos expression and neuronal marker NeuN suggests that all cells expressing c-Fos in response to administration of AngII are neurons, and quantification of the percentage of c-Fos-positive NeuN-positive cells reveals that 34.5% ± 1.9% of neurons are activated by the administration of Ang II (Figure 7G).

### Distribution of Neurons Responsive to Hypertonic NaCl Solution

Previous studies have shown that SFO contains osmosensory neurons that are activated by hypertonicity (Oldfield et al., 1991, 1994; Giovannelli and Bloom, 1992; Smith and Day, 1995; Anderson et al., 2000). *In vitro* electrophysiological studies using isolated SFO neurons have reported that a fraction of SFO neurons are activated by hypertonic stimulus (Anderson et al., 2000), and an intravenous infusion of hypertonic NaCl triggers expression of c-Fos in the SFO (Oldfield et al., 1991). Since these c-Fos studies commonly used a single coronal brain section of the SFO of male animals, we characterized the distribution of osmosensory neurons in the SFO of both males and females (four male and two female rats) by injecting them subcutaneously with 1 M hypertonic NaCl or control isotonic saline (0.9% NaCl). Serum osmolarities in saline- and hypertonic solution-injected animals were 302 ± 0.9 mOsm/kg and 333 ± 4.1 mOsm/kg, respectively (Figure 7F). To examine the distribution of neurons sensitive to hypertonic NaCl throughout the SFO, c-Fos immunoreactivity was analyzed in serial SFO coronal sections. As illustrated in Figure 7, c-Fos-positive neurons were scattered throughout the entire rostro-caudal extent of the nucleus, and this expression was specifically related to hypertonic NaCl, since animals injected with isotonic solution did not show any c-Fos-positive staining (Figure 7A). Consistent with previous reports, c-Fos positive cells were relatively sparse and found predominantly in the outer shell region, in both rostro-lateral and ventral shell areas (Figures 7D,E). This pattern was found across the entire rostro-caudal axis of the SFO. The density and the localization of hypertonic NaCl-sensitive neurons were similar in male and female rats. Analysis of co-localization between c-Fos expression and neuronal marker NeuN suggests that all the cells expressing c-Fos in response to administration of hypertonic NaCl are neurons, and quantification of the percentage of c-Fos-positive NeuN-positive cells revealed that only 13.3% ± 1.7% of neurons are activated by the administration of the hypertonic NaCl (Figure 7G).

## Discussion

Subfornical organ, which is one of the brain’s sensory circumventricular organs lacking the BBB, is a privileged site for communication between the peripheral circulation and the central nervous system, where brain cells can monitor blood-borne signals and affect physiological and pathological states (Dempsey, 1968). SFO is located in the anterior dorsal wall of the third ventricle at the confluence of the two interventricular foramina and the third ventricle, and adjacent to the choroid plexus (McKinley et al., 2003). The SFO is a highly vascularized structure featuring capillaries built of fenestrated endothelial cells (Sposito and Gross, 1987). This allows for the bidirectional movement of polar molecules between the hemal and neural environments of the SFO, resulting in the exposure of SFO neurons to systemic circulating stimuli. Like other CVOs, in addition to ependymocytes, the ventricular wall of the SFO contains tanycytes, which are specialized ependymal cells with oval morphology and lacking cilia. Tanycytes are connected by tight junctions that restrict the passage of circulating molecules from the SFO into the cerebrospinal fluid of the ventricle (Krisch et al., 1978; McKinley et al., 1990; Petrov et al., 1994; Langlet et al., 2013b). Thus, the BBB is shifted from the level of the capillary endothelium to the ventricular wall of the SFO. The SFO contains a dense population of neurons which have extensive afferent and efferent neural connections to other brain areas (McKinley et al., 2003, 2019). Accordingly, SFO has been shown to be involved in numerous functions requiring coordination between the central nervous system and the periphery, including energy metabolism (Pulman et al., 2006; Smith et al., 2009), thirst (Hollis et al., 2008), reproduction (Summerlee et al., 1987; Sunn et al., 2002), immune responses (Takahashi et al., 1997), cardiovascular regulation (Cancelliere and Ferguson, 2017; Jeong et al., 2019; Rossi et al., 2019), and systemic osmoregulation (Oldfield et al., 1994).

One of the most important functions of the SFO is its role in mediating the dipsogenic and pressor effects of circulating Ang II (Bickerton and Buckley, 1961; Simpson and Routtenberg, 1973; Hoffman and Phillips, 1976; Simpson et al., 1978; Ferguson and Bains, 1997). Together with the organum vasculosum of the lamina terminalis (OVLT), SFO has also been postulated to play an important role in the monitoring and relaying of osmotic and natremic information to other sites (Johnson and Gross, 1993; Smith and Ferguson, 2010).

Although many studies have reported neuronal c-Fos expression in this area in response to circulating Ang II and increased osmolarity, our study provides a more comprehensive understanding of the anatomical organization of different cellular populations within the rat SFO in both sexes. It also provides a more complete analysis of the BBB permeability of different subregions of the SFO in relation to the location of fenestrated and non-fenestrated vasculature, distribution of ependymal and glial cells, as well as of Ang II- and hypertonic NaCl-sensitive neurons.

### SFO Vasculature and BBB Permeability

Earlier studies used electron microscopy to analyze the distribution of capillaries with different morphology and defined several SFO subdivisions: rostral, transitional, central and caudal subregions, with dorsal, ventromedial and lateral zones of the latter three sub-regions (Sposito and Gross, 1987; Shaver et al., 1990). More recent studies analyzing neural connectivity, receptor binding, and functional neuroanatomy using *c-fos* expression simplified this division to two regions: “outer shell” and “ventromedial core” (McKinley et al., 2003; Oldfield and McKinley, 2015). In the present study, we have combined a number of approaches to correlate the analyses of the distribution of different types of vasculature and vascular permeability with functional neuroanatomy using c-Fos, as well as examinations of multiple glial subtypes.

Our analysis of permeability marker EB shows that SFO is an ovoid midline structure that extends ∼500 μm in the rostro-caudal axis, 800 μm in the lateral axis, and 600 μm in the dorsal-ventral axis (Figure 1). We observed that the distribution of EB signal differs between the rostral, medial and caudal regions of SFO (Figure 1). While EB fills the entire volume of the SFO, the BBB permeability is not uniform throughout the SFO. The rostral region of SFO is less permeable to EB, and the permeability increases in the medial part of the nucleus, remaining the greatest through the medial and caudal regions. In addition, the dorsal aspect of the SFO that lies along the ventral border of the hippocampal commissure shows reduced BBB permeability (Figure 1). These findings are corroborated by our observations showing that non-fenestrated vessels occupy the dorsal and the lateral borders of the entire nucleus. Moreover, while the rostral part of the SFO harbors a dense vascular network comprised of large vessels and small capillaries, none of these endothelia appear to be fenestrated (Figure 2). This vascular pattern delineates a continuous external zone with reduced BBB permeability that begins at the rostral wall of the SFO, extends through the dorsal, ventral, and lateral borders of the nucleus in the rostro-caudal direction, and terminates at the most caudal part of the SFO, where the nucleus merges with the choroid plexus. Thus, the outer shell resembles a 3-dimentional baseball glove-like-shaped external zone of the SFO that includes the rostral, dorsal, and lateral outer parts of the nucleus (Figure 1). This area has reduced BBB permeability, and is occupied by an extensive network of non-fenestrated vasculature comprising large vessels (up to ∼100 mm in diameter) and smaller capillaries (5–10 mm in diameter).

The ventromedial core appears only in medial to caudal portions of the SFO and runs until it fuses with the choroid plexus at the caudal pole of the nucleus (APB −0.96 to −1.20). Consistent with previous reports, our data show that the ventromedial core features a very dense vascular network comprised of multiple convoluted capillary loops made of fenestrated endothelium. Accordingly, our data show that this area had the greatest BBB permeability (Figures 1, 2).

The presence of the fenestrated vasculature enabling blood-borne signals to penetrate the SFO parenchyma and interact with local neurons is the most unique feature of CVOs. Our data illustrate an extremely dense and complex network of both fenestrated and non-fenestrated vasculature expending throughout the entire SFO (Figure 2 and Supplementary Video 1). Due to this high density of the vascular network, the blood flow within SFO was estimated to be doubled and blood-to-tissue transfer many orders of magnitude greater than in the adjacent brain areas (Gross, 1991); a feature essential for the SFO functioning as a sensory CVO (Gross, 1991; McKinley et al., 2003).

Interestingly, we found that the same blood vessel can be non-fenestrated in one SFO subregion and contain fenestrations in another part. For example, the subfornical artery lacks fenestrations, and only once it branches into smaller capillaries, becomes fenestrated (Supplementary Video 1). These observations suggest that the presence or lack of fenestra might not be due to the allocation of distinct vascular types to a specific subregion, but possibly determined by the immediate environment or factors found (e.g., secreted) locally near the endothelial cells, consistent with the concept that the brain microenvironment regulates endothelial cell features and BBB properties (Stewart and Wiley, 1981; Sabbagh et al., 2018; Villabona-Rueda et al., 2019). Thus, it is conceivable that local neurons or glia cells provide signals to the endothelial cells to determine their features and the presence of fenestra, e.g., by secreting vascular factors [e.g., VEGF (Cao et al., 2004; Langlet et al., 2013a)], to locally regulate the BBB.

### Glia Cell, Ependymocytes, Tanycytes, and Pericytes

Our study reveals that SFO harbors multiple non-neuronal populations and characterizes the distribution and organization of tanycytes, ependymocytes, astrocytes, NG2 glia, and pericytes within the SFO. These diverse cell types typically lack a distinct cellular marker that allows to identify them. For example, GFAP is expressed by astrocytes and a fraction of tanycytes, vimentin is expressed by all tanycytes and ependymocytes, and NG2 is expressed by pericytes and NG2 glia cells. Thus, in addition to expression of cellular markers, the identification of these populations requires a consideration of cell morphology and position.

As in other CVOs, we found vimentin-positive cells located at the dorsal part of the SFO and lining the third ventricle (Figures 3, 4). In the rostral part of the SFO, the ventricular wall contains vimentin-positive cells that displayed a typical ependymocyte morphology, a cuboidal shape cell organized in a single layer and displaying intense vimentin signal surrounding a prominent round and centrally located nucleus. In medial sections of the SFO, the ependymal cells are gradually replaced by vimentin-positive cells that exhibited typical tanycyte morphology: an elongated oval soma with apical processes extending into the parenchyma (Figures 3, 4). Notably, the cell bodies of SFO tanycytes exhibit not only a more round shape as compared to ependymocytes, but also express lower levels of vimentin. The fine processes of tanycytes create an interweaved network within the ventromedial core of the SFO (Figures 3B,C, 4A) and project toward SFO capillaries (Figures 4D–F). This interweaved network of tanycytes appears as two distinct populations: a ventromedially-located population showing immunofluorescence for vimentin and GFAP, and a laterally-located population expressing vimentin but not GFAP (Figures 3B,C). The laterally-located population has more pronounced vertical processes extending into the SFO parenchyma, similarly to the organization of tanycyte processes found in other CVOs such as the OVLT and the median eminence (Langlet et al., 2013b; Prager-Khoutorsky and Bourque, 2015). Conversely, SFO tanycytes, located in the ventromedial SFO, display shorter and more convoluted processes. Our observations suggest that the lateral population of tanycytes mostly contact large non-fenestrated blood vessels located in the outer shell, while ventromedial tanycyte processes project toward small fenestrated capillaries located in the ventromedial core. However, whether these morphologically distinct tanycyte subtypes serve different function remains to be determined.

In addition to ependymocytes and tanycytes, the SFO comprised additional glia types. Similarly to the non-CVO areas with complete BBB, we found GFAP-positive and vimentin-negative astrocytes (Figures 3A–C). These astrocytes feature classical stellate astrocyte morphology and are very abundant in the outer shell, where dense astrocytic processes create an extensive network surrounding non-fenestrated large blood vessels, putative venous vasculature feeding the septal veins. Interestingly, despite being non-fenestrated, these large vessels differ from BBB-complete vessels found outside the SFO, since in addition to astrocytic endfeet, they are also contacted by vimentin-positive processes (Figure 3E). Conversely, fenestrated capillaries located in the ventromedial core are surrounded by tanycyte processes expressing mostly vimentin (Figure 4F). This observation is consistent with a previous electron microscopy study that indicated that fenestrated capillaries located within the ventromedial core of the SFO are encompassed by several layers of tanycyte processes (Krisch et al., 1978). A similar arrangement has been documented in the median eminence, where tanycytes lining the floor of the third ventricle send long processes that form end-feet structures that wrap the capillaries of the pituitary portal system (Prevot, 2002), as well as in the OVLT, where fenestrated vessels are contacted by vimentin-positive processes arising from the tanycytes forming the rostral wall for the third ventricle (Prager-Khoutorsky and Bourque, 2015). The interaction of tanycyte endfeet with fenestrated vasculature may play a key role in the local transport and diffusion of blood-borne molecules into the parenchyma (García et al., 2003; Bolborea and Dale, 2013; Balland et al., 2014; Anesten et al., 2017; Pasquettaz et al., 2020).

An additional glia type found in the SFO is NG2 glia, or oligodendrocyte precursor cells (OPCs). While NG2 glia display complex, highly branched morphology, and resemble protoplasmic stellate astrocytes, they do not express GFAP and vimentin (data not shown), as well as other astrocytic markers such as S100b (Nishiyama et al., 2002). Recent studies suggest that NG2 glia represent a functionally distinct cell population with multipotent self-renewal potential and are capable of differentiating not only to oligodendrocytes, but also to neurons and astrocytes (Du et al., 2020). Further characterization of NG2 glia will be required to understand the function of these cells in the SFO.

Lastly, we found a previously undescribed population of pericytes located mostly in the ventromedial SFO core. The density of vessels covered by pericytes varies throughout the brain (Dermietzel et al., 2006; van Dijk et al., 2015). Our observations suggest that pericytes create a dense network around the SFO capillaries. Notably, in the BBB-complete areas, pericytes are found between the endothelial cells and astrocytic end feet, forming a structure critical for the maintenance of intact BBB, where loss of pericytes is associated with BBB leakiness (Uemura et al., 2020). Remarkably, in the SFO, we found that fenestrated vasculature that features an incomplete BBB and lack astrocyte endfeet is encased in a scaffold created by pericytes.

### Distribution of Neurons Responsive to Ang II and Hypertonic NaCl

Previous studies imply that the topographic distribution of neurons within the SFO is important as it determines their efferent projections and thus defines the downstream target brain region and physiological functions (McKinley et al., 2015). For example, neurons located in the outer shell project to areas associated with body fluid homeostasis such as magnocellular neurosecretory cells of the supraoptic and paraventricular nuclei, OVLT, median preoptic nucleus, and prefrontal cortex. Neurons located in the ventromedial core project to areas associated with autonomic control, such as the parvocellular division of the paraventricular nucleus and the bed nucleus of the stria terminalis (McKinley et al., 2003, 2015). Since some of the key functions of the SFO are to mediate the central effects of Ang II and sense changes in blood osmolality, characterization of the topographic distribution of neurons activated by Ang II and hypertonicity is critical for understanding complex roles of the SFO in regulating neural pathways involved in thirst and water intake, salt appetite, vasopressin release, pressor responses and autonomic functions.

Our study reveals that a subset of neurons responsive to hypertonic NaCl are located mainly in the external zone of the SFO, which includes the outer shell including the ventral perimeter of the SFO. While this localization of Na^+^- osmo-sensitive neurons is consistent throughout the rostro-caudal axis of the nucleus (Figures 7D,E), osmosensitive neurons are more abundant in the rostral as compared to the caudal SFO (Figure 7H, *p* < 0.008). Previous studies of c-Fos immunoreactivity in response to osmotic stimulation showed similar findings (Oldfield et al., 1991; Smith and Day, 1995). Notably, we found that only ∼13% of neurons were responsive to hypertonic NaCl, in contrast to our previous study evaluating the distribution of hypertonic NaCl neurons in the OVLT, showing that about half of neurons were activated by this stimulus (Prager-Khoutorsky and Bourque, 2015). Importantly, distribution of c-Fos in response to hypertonicity has been commonly evaluated only in male rats. Our analyses were conducted on both sexes, revealing no significant differences in the number and distribution of hypertonic-NaCl-responsive neurons between SFO of male and female rats. Previous studies have shown that neurons located in the outer shell have direct as well as indirect (*via* the median preoptic nucleus and the OVLT) efferent projections to magnocellular neurons of the supraoptic and paraventricular nuclei to regulate vasopressin secretion (Oldfield et al., 1994); and have polysynaptic thalamocortical pathways driving thirst (Hollis et al., 2008). Further work is required to establish if hypertonic NaCl responsive neurons located in the outer shell exhibit identical efferent pathways in both sexes.

As with the hypertonic NaCl stimulation, studies analyzing c-Fos expression to identify Ang II-responsive neurons have commonly used only male animals and illustrated their findings using a single coronal tissue section. These studies demonstrated that while intravenous infusion of Ang II stimulates c-Fos expression in neurons throughout the SFO (McKinley et al., 1992), low physiological concentrations of Ang II increases c-Fos expression mostly in the ventromedial core (McKinley et al., 1992). Likewise, this region was shown to have a higher density of Ang II receptors (Lenkei et al., 1995, 1997; McKinley et al., 1998; Giles et al., 1999). Our analysis of rostral and medial sections of the SFO from male rats corroborate these findings, showing that most neurons expressing c-Fos following a systemic bolus of Ang II are located in the ventromedial core (Figure 7B). However, we found that in the caudal aspect of the SFO, a large fraction of outer shell neurons is also activated by systemic Ang II (Figure 7C). Notably, in contrast to hypertonic NaCl-sensitive neurons, whose number decreases from the rostral to caudal SFO, the number of Ang II-activated neurons is significantly larger in the caudal area (41.1% ± 2.4%) as compared to the rostral SFO (28.9% ± 3.4%) (Figure 7H, *p* < 0.025). Our data indicate that these two populations overlap in the outer shell of the caudal SFO. Future studies should determine whether hypertonic NaCl-sensitive and Ang II-responsive neurons might represent two distinct neuronal populations located in the same region. However, it is plausible that the caudal region of SFO may contain neurons responding to both hypertonic NaCl and Ang II.

Surprisingly, we found that the distribution pattern of neurons activated in response to systemic Ang II was different in SFO sections from female rats (Figures 7D,E). Our data analyzing female SFO suggest that in the caudal region, Ang II-responsive neurons are located predominantly in the outer shell, where the majority neurons express Ang II-induced c-Fos. We found that in contrast to males, only a small fraction of ventromedial core neurons is weakly activated by systemic Ang II in females (Figure 7E), suggesting that the caudal part of the SFO might contain sexually dimorphic neuronal populations. Further work is required to establish the pattern of neuronal efferent projections in females and study if the caudal subregion of the SFO contains distinct populations of neurons in females that are not found in males. The differential organization of Ang II-responsive neurons in males and females suggests that in addition to sex differences in the peripheral renin-angiotensin system (Tatchum-Talom et al., 2005; Xue et al., 2005), the central Ang II-mediated functions may vary between sexes.

In addition to osmolarity and Ang II, previous studies reported that different SFO sub-regions contain neurons that vary in their ability to respond to a number of humoral stimuli, as well as in their pattern of connectivity to other brain areas. For example, experiments involving c-Fos detection have shown that increases in the circulating levels of the hormone relaxin preferentially activate neurons in the outer shell (McKinley et al., 1997, 1998), spatially coinciding with hypertonic NaCl responsive neurons. In addition to relaxin and NaCl, previous studies also showed that outer shell contains calretinin- and cholinergic-containing neurons (Jhamandas et al., 1989; Huang et al., 2019), whereas calbindin-containing neurons are predominantly found in the ventromedial core (Huang et al., 2019), coinciding with Ang II responsive neurons. Future studies should examine if these neuronal populations located in the outer shell show similar distribution in both sexes.

Recent studies focusing on the role of SFO in fluid intake in mice identified a few neuronal subpopulations: thirst-driving excitatory neurons that express the transcription factor ETV1 and nNOS; and inhibitory vesicular GABA transporter (VGAT)-expressing neurons that suppress drinking (Leib et al., 2017; Augustine et al., 2018). Most excitatory neurons are activated upon dehydration, showing a robust c-Fos expression after water deprivation (Betley et al., 2015; Oka et al., 2015; Augustine et al., 2018; Pool et al., 2020). A recent study also demonstrated that these excitatory dehydration-activated neurons consist of two non-overlapping populations: Rxfp1-expressing neurons that are activated by hypertonic NaCl to trigger osmotic thirst (water intake), and prodynorphin-expressing neurons that are activated by hypovolemia, a condition that increases endogenous Ang II levels, to trigger volemic thirst (water and saline intake) (Pool et al., 2020). Whether Rxfp1- and prodynorphin-expressing excitatory neurons described in mice correspond to hypertonic NaCl and Ang II responsive neurons, respectively, such as described in the current and previous studies in rats, remains to be elucidated. Notably, diverse populations of excitatory and inhibitory neurons do not show specific topographic localization to either the outer shell or the ventromedial core, but rather distributed throughout the SFO. Future studies should establish whether inhibitory neurons are also activated by these stimuli and how specific combinations of different neuronal subtypes are activated by distinct physiological states to mediate complex homeostatic responses.

### Concluding Remarks

Although our study reveals that the majority of cells expressing c-Fos after stimulation with hypertonic saline and Ang II are NeuN-positive neurons (Figure 7), it is possible that a fraction of non-neuronal cells (e.g., astrocytes or tanycytes) also express c-Fos in response to these stimuli. Further work is required to establish the potential involvement of diverse populations of non-neuronal cells in the SFO *in vivo*. The SFO lacks a conventional BBB and, thus, allows local neurons to detect circulating molecules and direct adaptive central responses to peripheral signals. Our study provides a comprehensive description of the spatial organization of the rat SFO and describes subregions featuring different types of vasculature (fenestrated and non-fenestrated) and showing various degrees of BBB permeability. In addition, our study provides a detailed description of the location and interrelationships between different cell types within the borders of the SFO. Notably, we highlight the presence of a population of tanycytes whose densely interweaved processes are distributed throughout the SFO, embedding local vasculature and neurons. Tanycytes, pericytes, and other glia types (astrocytes, NG2 glia), may play an important role in the structural and metabolic support of local neurons and the regulation of their electrical activity. Pericytes and tanycytes may play an important role in the regulation of the BBB at the fenestrated capillaries to control the access of peripheral molecules into the SFO.

## Data Availability Statement

The original contributions presented in the study are included in the article/Supplementary Material, further inquiries can be directed to the corresponding author/s.

## Ethics Statement

The animal study was reviewed and approved by Comparative Medicine and Animal Resources Centre of McGill University.

## Author Contributions

A-IH and MP-K designed the experiments and wrote the manuscript. A-IH, SK, SZ, and JY performed the experiments. All authors contributed to the article and approved the submitted version.

## Conflict of Interest

The authors declare that the research was conducted in the absence of any commercial or financial relationships that could be construed as a potential conflict of interest.

## Publisher’s Note

All claims expressed in this article are solely those of the authors and do not necessarily represent those of their affiliated organizations, or those of the publisher, the editors and the reviewers. Any product that may be evaluated in this article, or claim that may be made by its manufacturer, is not guaranteed or endorsed by the publisher.

## Supplementary Material

Supplementary Figure 1 Analyses of Evans blue, number of neurons, and AngII-sensitive neurons in rostro-caudal axis. **(A)** Plots show mean ± SEM Evan Blue (EB) fluorescence in rostral, medial, and caudal SFO from six rats, analyzed by two-way ANOVA with Tukey’s multiple comparison test. **(B)** Distribution of NeuN-positive neurons in the rostro-caudal axis of the SFO from 14 rats shown as mean ± sem, analyzed by Kruskal–Wallis test followed by Dunn’s multiple comparison. **(C)** Plots show mean ± SEM percentage of c-Fos-positive neurons in SFO of rats injected with 2 mg/ml AngII in male (*n* = 4) and female (*n* = 4) rats, analyzed by a two-tailed, unpaired, parametric Student’s *t* test. **(D)** Plots show mean ± SEM percentage of c-Fos-positive neurons in the shell region of rostral, medial, and caudal SFO in male (*n* = 4) and female (*n* = 4) rats injected with 2 mg/ml AngII, analyzed by a two-tailed, unpaired, parametric Student’s *t* test. *P* values are indicated above each plot, ^∗^*p* < 0.05, ^∗∗^*p* < 0.01, ns not significant.

Supplementary Video 1 Organization of fenestrated and non-fenestrated vasculature in the SFO. Consecutive coronal SFO sections spanning 300 mm in the rostro-caudal direction immunolabeled for a pan-endothelial marker (RECA1, green) and a marker of fenestrated vasculature (PV1, red). Fenestrated capillaries occupy the core of the medial and caudal SFO regions, while non-fenestrated vessels are located in the rostral area and creating a 3-dimentional shell around the SFO core. A non-fenestrated subfornical artery emerges in the middle of the SFO core from the caudal aspect.
