# Supplementary figures and images for: Anatomical Organization of the Rat Subfornical Organ
Source: Front Cell Neurosci. 2021 Sep 6;15:691711. doi: 10.3389/fncel.2021.691711 (PMC8450496; doi:10.3389/fncel.2021.691711)

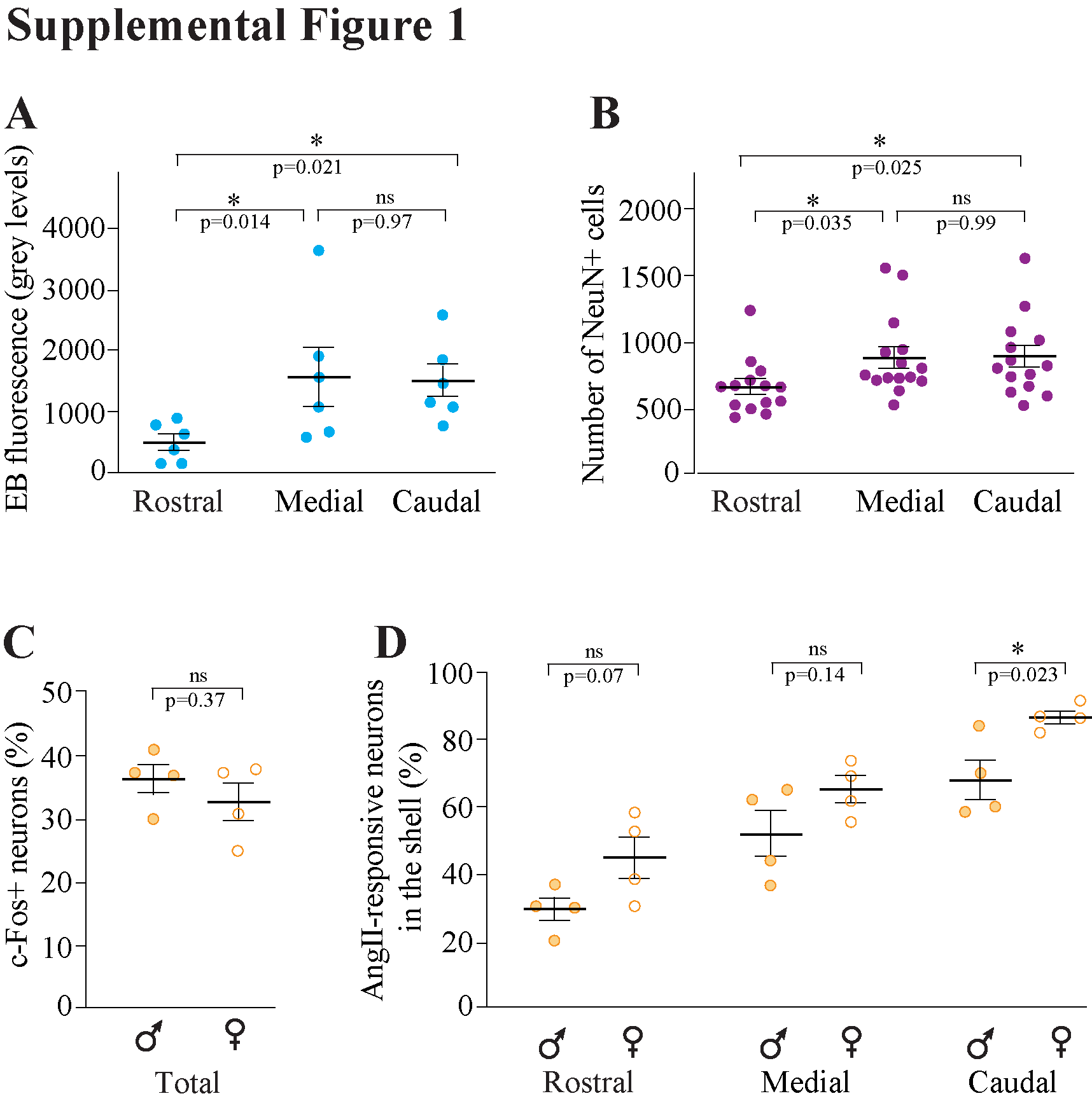

Supplement: Supplementary Figure 1 — Analyses of Evans blue, number of neurons, and AngII-sensitive neurons in rostro-caudal axis. (A) Plots show mean ± SEM Evan Blue (EB) fluorescence in rostral, medial, and caudal SFO from six rats, analyzed by two-way ANOVA with Tukey’s multiple comparison test. (B) Distribution of NeuN-positive neurons in the rostro-caudal axis of the SFO from 14 rats shown as mean ± sem, analyzed by Kruskal–Wallis test followed by Dunn’s multiple comparison. (C) Plots show mean ± SEM percentage of c-Fos-positive neurons in SFO of rats injected with 2 mg/ml AngII in male (n = 4) and female (n = 4) rats, analyzed by a two-tailed, unpaired, parametric Student’s t test. (D) Plots show mean ± SEM percentage of c-Fos-positive neurons in the shell region of rostral, medial, and caudal SFO in male (n = 4) and female (n = 4) rats injected with 2 mg/ml AngII, analyzed by a two-tailed, unpaired, parametric Student’s t test. P values are indicated above each plot, ∗p < 0.05, ∗∗p < 0.01, ns not significant. [file Image_1.TIF]
